# A cross-national study of family-friendly policies, gender egalitarianism, and work–family conflict among working parents

**DOI:** 10.1371/journal.pone.0291127

**Published:** 2023-09-20

**Authors:** Hsinyi Hsiao

**Affiliations:** Department of Social Work, Tzu Chi University, Hualien, Taiwan; University of Zaragoza: Universidad de Zaragoza, SPAIN

## Abstract

Social policies impinge on daily lives of individuals and affect how they negotiate work and family demands. To fill in the void in the international work–family literature regarding whether public family-friendly policies effectively decrease work–family conflict the present study examined multilevel effects of family-friendly policies, organizational type, and family characteristics on working parents’ work–family conflict by gender using random samples of 6,878 individuals in 24 countries in Africa, Asia, Europe, and North and South America. Drawn on role stress theory, gender egalitarianism, and institutional theory this study showed that parental leave policies have greater influence on work–family conflict among men compared to women. Individual dimensions of parental leave policies on men’s experience of work–family conflict impinged on workplace characteristics and family characteristics. Implementing parental leave policies with high flexibility and higher rates of income replacement may help men with working spouses or who are employed in the public sector to reduce bidirectional conflicts between work and family. Women generally were not protected by individual dimensions of parental leave policies. Instead, societal attitudes towards gender played a key role in helping women reduce bidirectional conflicts between work and family roles.

## Introduction

Increasing interdependence among economies and the rise in women’s labor force participation have led to an obvious trend of decreasing fertility rates in both Western and Eastern countries. The reduction mostly happened during the 1990s for Western countries and during the 2000s for East Asian countries. For example, the fall in fertility rate was particularly steep in South Korea and Taiwan [1]. With the lowest fertility rate among Organisation for Economic Co-operation and Development (OECD) countries, South Korea’s fertility hit a record low of 1.08 in 2005 [1, 2] and has continued to decline into 2022 [3]. Female labor force participation and low fertility rates have a lot to do with personal choices and preferences.

Both individual and macro factors at the country level (i.e., cultural, institutional, and economic factors) contribute to the work–family interface experience such as competing demands and thus, conflicts [4, 5]. The importance of both micro and macro contributing factors to our work and family experiences calls for refined examination of our work–family relationship in cross-national contexts. Importantly, individuals work under country-specific legal and normative constraints that affect how they negotiate work and family demands. However, national policies could make such constraints and negotiation easier or more difficult for parents. For example, to address the decreasing fertility rate and conflicts experienced by employed parents in negotiating work–family demands, many countries have provided family-friendly policies to allow working parents to take time off to fulfill familial responsibilities in the hopes of alleviating work–family conflict [6, 7].

Most policy studies have used qualitative methods to examine various forms of parental leave policies across countries, making it challenging to draw cross-national comparisons of the generosity of individual dimensions of policies [6, 8, 9]. Some scholars used quantitative methods to examine the effects of parental policies on maternal and child health [10] and the gender division of household work [11–13], rather than decreasing work–family conflict. Other scholars used cross-national data (e.g., European Social Survey) to investigate the effects of institutional or cultural effects on work–family conflict [4, 14, 15]. Most cross-national research has investigated work–family conflict separately at micro (individuals and families) or macro (social policies such as parental leave and benefits) levels. Although the objective of family-friendly leave policies is to promote work–life balance, little is known about the effectiveness of these policies in the context of work–family conflict [15].

The extant research has shown that gender and family characteristics play key roles in shaping individual perceptions toward work–family conflict [16]. However, findings of the literature examining gender differences in work–family conflict have been inconsistent. Some scholars found that men experience greater work-to-family conflict than women [17, 18], but other studies found that women report higher levels of work-to-family conflict than men. Discrepancies in the results of the literature examining the effects of gender egalitarianism and family characteristics on work–family conflict could be attributed to differences in methodologies (e.g., only managerial employees, number of participating countries).

In their meta-analytic findings derived from reviewing 332 studies published between 1992 and 2018, Allen et al. [4] underscored the need for future studies to examine institutional policies as a factor that could explain between-country differences in work–family experiences in the context of culture and economy. Thus, recognizing the gaps in cross-cultural research, the present study sought to understand: (a) generosity of national family-friendly leave policies by comparing individual dimensions of policies (i.e., flexibility of use, length of leave, income replacement rate) across countries; (b) whether various dimensions of family-friendly leave policies effectively decrease work–family conflict; (c) whether gender egalitarianism decreases work–family conflict; and (d) how the relationship among organizational type, family characteristics, and work–family conflict varies by family-friendly leave policies and gender egalitarianism.

Drawing on role theory [19], the GLOBE study’s gender egalitarianism [20], and institutional theory [21], the present study used International Social Survey Program (ISSP) data collected in 2005 from 6,878 random samples of working parents in 24 countries in Africa, Asia, Europe, and North and South America to examine multilevel effects of family-friendly policies and gender egalitarian on working parents’ work–family conflict by gender. Due to inconsistencies regarding the moderating effects of gender in the literature, the present study performed hierarchical linear modeling analysis separately by gender rather than including it as a control variable.

Using cross-national probability random samples and a quantitative approach, the present study was among the first to examine the effectiveness of individual dimensions of national family leave policies on work–family conflict and more thoroughly explore similarities and differences between fathers and mothers. This study shows that parental leave policies had greater influence on work–family conflict among fathers compared to mothers. Using ISSP data in 2005, although collected more than a decade ago, can shed light on the trend of decreasing fertility rates between 1990 and 2005 experienced by East Asian countries relevant to low flexibility of parental leave (e.g., South Korea) and low income-replacement rates (e.g., Taiwan). For countries with low fertility rates and increasingly aging populations in 2022, the results provide important implications for policy makers when implementing family-friendly policies to promote work–family balance. Namely, the generosity of individual dimensions of policies such as flexibility of use, length of leave, and income replacement rate are critical to address concerns relevant to use of parental leave and work–family conflict, especially in the United States, the only developed country that has not offered a national paid leave policy as of 2022 [22].

Moreover, individual dimensions of parental leave policies on men’s experience of work–family conflict hinge on workplace characteristics (e.g., organization type) and family characteristics (e.g., having a working spouse with full-time employment). Hence, given the influence of globalization with goods manufacturing being outsourced to overseas suppliers, the results provide guidance for policy makers and employers in public and private sectors in developing countries (e.g., India, Vietnam) featuring an increasing rate of labor force participation among women, traditional gender attitudes, inflexible work environments (e.g., electronics and garment factories), and family structures similar to those of the past decades in developed countries. The findings of this study also further the improvement of family friendly policies, development of work–family theoretical models and meta-analytic research in cross-cultural contexts by identifying important factors in institutional environments (e.g., public organizations) and gender in the context of diffusion and patterns of parental leave policies.

### A theoretical perspective of work–family conflict

Much of the research on work and family issues has relied on the occupational stress perspective and role theory [19]. According to role theory, roles are the result of the expectations of others about the appropriate behavior of someone in a particular position. When various members of the role set hold different role expectations for the focal person, they may impose pressures that result in psychological conflict. WFC has been defined as “a form of interrole conflict in which the role pressures from the work and family domains are mutually incompatible in some respect” [23] and consists of two broad dimensions: work-to-family conflict (hereafter WFC; i.e., work interfering with family) and family-to-work-conflict (FWC; i.e., family interfering with work) [24]. Drawn on role theory, time is a finite resource that cannot be expanded by engaging in multiple roles, an hour devoted to work represents an hour that will not be available in the family domain. The competing concept between work and family roles suggests that when people devote longer hours to work, they are less likely to meet family demands. Thus, work or family domains can impinge on each other, and interference will be experienced in two ways: bringing work home or life outside of work negatively affecting work performance.

WFC has been treated as an outcome of stress. On the other hand, it often has been considered a potential source of stress that has adverse effects on affective work outcomes such as organizational commitment [25–27], job satisfaction [4, 28, 29], and intention to quit [30]. Moreover, family characteristics (e.g., marital status, parental status, having a working spouse) may also lead to higher levels of FWC [31]. Individuals who are married and have children typically have more family role responsibilities. When confronted with antecedents such as work domain stressors, married parents may experience greater WFC than those who are single or without parental responsibilities [32].

### Gender egalitarianism, family characteristics, and work–family conflict

Understanding the gender-role attitudes (traditional or egalitarian) of individuals is critical to predicting how much conflict they experience in negotiating work and family demands. From the perspective of social role theory [33], gender roles have been considered as “stereotypes produced by the sexual division of labor and societal expectations and through socialization and the formation of gender roles, the behaviors of men and women generally support and sustain the division of labor” [34]. Korabik et al. [35] posited that women with traditional gender-role attitudes might experience higher levels of WFC if they have paid employment, compared to those with more egalitarian attitudes about work and life. Similarly, men with traditional gender-role attitudes might experience higher levels of FWC if they spend time taking care of children or performing domestic tasks, compared to those with more egalitarian attitudes.

Based on GLOBE’s study, gender egalitarianism, referring to how societies divide roles between women and men, is generally conceived of as a precursor to WFC [7, 16, 20]. The more gender egalitarian a society, the less it relies on biology to determine gender roles. Using random samples collected from 10 European countries that participated in the European Social Survey in 2005, Fahlén [16] found that a greater gender gap in WFC perceptions among individuals in countries with weaker support of work–family policies and more traditional gender norms. Similarly, Rajadhyaksha and Velgach [36] found that in India, individuals with more traditional ideologies experienced more WFC. Moreover, gender had moderating effects on the relationship between gender ideology and FWC. Women with traditional attitudes experienced greater FWC than egalitarian women and men and traditional men. A comparative study by Poelmans et al. [37] examined the relationship between gender egalitarianism and WFC among 835 parents with full-time jobs in Canada, Spain, Taiwan, and the United States. Findings suggested that parents with traditional gender-role attitudes experienced greater WFC than those with egalitarian attitudes. Ruppanner and Huffman’s [7] multilevel study using ISSP data found that women’s empowerment at the country level decreased mothers’ FWC, whereas fathers in more gender-empowered countries tended to have higher levels of WFC. Thus, the first hypothesis was as follows:

H1: Parents in countries with more egalitarian gender practices will report lower levels of WFC.

Gender egalitarianism and family characteristics are generally conceived of as a precursor to work–family conflict. Family characteristics (e.g., marital status, having a working spouse) may associate with bidirectional work–family conflict [31]. Individuals who are married and have children typically have more family role responsibilities. When confronted with antecedents such as work domain stressors, married parents may experience greater WFC than those who are single or without parental responsibilities [32]. Fathers in dual-earner couples experienced significantly greater WFC than mothers, but other studies have found that women report higher levels of WFC than men [17]. In addition, family characteristics may lead to higher levels of FWC [31]. However, previous studies showed mixed results, which might be related to inconsistency in samples and measurements. It remains unclear whether gender egalitarianism moderates the relationship between family characteristics and work–family conflict. Thus, the second hypothesis was as follows:

H2. The association between family characteristics (i.e., marital status, having a spouse with full-time employment) and parents’ work–family conflict will be weaker in countries with more egalitarian gender roles.

### Family-friendly policies and policy dimensions

Scholars have used institutional theory to investigate the diffusion of work–family practices among organizations [38, 39]. Institutional theory proposes that organizations are influenced by normative pressures arising from external sources such as the government [21]. National family-friendly policies are considered institutional factors that lead to consequences and norms for employers and workers to encourage conformity in terms of work such as parental leave following the birth or adoption of a child [40]. Thus, working parents in most countries today are entitled to different types of leave known by various labels. The most common types of leave are maternity, parental, and paternity leave. Maternity leave aims to protect working women during pregnancy and recovery from childbirth. By safeguarding employment and income security during and after maternity, maternity protection contributes to the health and well-being of women and their babies and the realization of gender equality and women’s empowerment [41]. Parental leave is gender-neutral, job-protected leave designed to allow employed parents to care for small children at home [42]. Parental leave has been considered an effective strategy for increasing involvement of women in the labor market, promoting gender equality and active participation in childcare, facilitating work–family reconciliation, and increasing the national fertility rate [43]. Paternity leave also allows men to spend a few days or weeks with family after childbirth or adoption with job protection [42].

Allen et al. [40] pointed out that it is challenging to compare national parental leave policies that govern work–family time due to differences such as time allotted for leave and percentage of salary paid. For example, parental leave provisions vary in terms of four main dimensions: (a) length of total continuous leave available (maternity, paternity, parental, and childcare leave; typically ranging from 9 months to 3 years and not always with pay); (b) entitlement—a mix of family and individual entitlements with the remainder divided equally between individuals (which may or may not be transferable); (c) income replacement—flat, means-tested, partial rates, or a combination of methods; and (d) flexibility, or how leave can be used (at times chosen by the employee, in one segment or shorter blocks of time, on a full-time or part-time basis, or the ability to take additional leave in special circumstances) [44]. Compared with the United States, which provides only 12 weeks of unpaid parental leave [9], Swedish parental leave offers the most generous combination of time and financial support to parents [45]. It provides 480 days of leave per child up to the age of 8 or until they complete their first year of school, a high income replacement rate (80%) for most of the leave period, and significant flexibility in terms of using leave in more than one block and on a part-time or full-time basis [9].

Various forms of national parental leave policies feature economic, cultural, and social differences [9]. Previous research has suggested that economic development is an important factor that may affect the work–family interface [46–49]. Stier et al. [15] and Allen et al. [40] used gross domestic product as an indicator of national economic development to compare WFC and FWC across countries by economic context. Economic prosperity has been viewed as a resource to lessen WFC, such as higher pay for workers to purchase groceries, and help manage work–family roles. Similarly, economic prosperity may influence organizations to offer family-friendly practices [40].

A wide variety of cross-national research has investigated the effects of parental policies on maternal and child health, well-being of preschool children and their parents, labor market attachment among women, and gender roles in families. Previous empirical studies using macro data from Organisation for Economic Co-operation and Development countries found that extended parental and maternity leave had positive effects on child health outcomes. Using data from 18 countries from 1969 to 2000, Tanaka [10]) found that the length of job-protected paid leave decreased infant mortality rates. Recent sociological research has studied the gender division of housework in terms of national context [11–13]. Using 44 time-use surveys between 1965 and 2003 from 20 countries combined with original national-level data, Hook [50] employed a multilevel model to examine the relationship between national context and household labor (e.g., laundry, caring for sick relatives) among men. He found that housework labor time among men increased with national levels of employment of women. The effects of children on men’s housework labor time depended on women’s national employment hours, the length of available parental leave, and the eligibility of men to take parental leave, indicating that particular public policies affect men in specific household situations. Campaña et al. [51] used data from the Harmonized European Time Use Survey and the European Values Study in the 2000s and 2010s to examine the gender gap in time allocation in European countries. They found that more generous paternity and parental leave policies imply that men may be more involved in care responsibilities, which may reduce the gender gap in unpaid work and potentially even paid work, because women could devote more time to their jobs.

Family-friendly policies are believed to ease the burden of negotiating work and family demands and lead to lower levels of WFC. The International Policy Center for Inclusive Growth and UNICEF in 2020 [52] reviewed maternity, paternity, and parental policies in the workplace among 24 countries in Latin America and the Caribbean. They found that extended leave policies not only increase the likelihood of breastfeeding but also improve mothers’ stress and gender equality. However, few studies have examined the effects of parental policies on WFC using cross-national random samples. Using ISSP data in 2002, Stier et al. [15] found that childcare availability and to a certain extent, maternity policies helped women and men reduce their sense of conflict. They suggested future studies use each policy’s measure to examine the effects of family-friendly policies across countries.

H3: Parents in countries with more family-friendly leave policy bundles—characterized by (a) higher flexibility of use, (b) longer length of leave, and (c) higher income replacement—will report lower levels of WFC.
H4: The association between family characteristics (i.e., marital status, having a spouse with full-time employment) and parents’ work–family conflict will be weaker in countries with more family-friendly leave policy bundles—characterized by (a) higher flexibility of use, (b) longer length of leave, and (c) higher income replacement.

### Organization type and WFC

Some organizational characteristics may moderate the relationship between family-friendly public policy bundles and work–family conflict. According to institutional theory, organizations are influenced by normative pressures arising from external sources such as the government [21]. Under some conditions, these pressures may cause organizations to be guided by legitimated environment [53]. During recent decades, governments have strongly encouraged employers to provide family-friendly programs, such as childcare and flexible scheduling [54]. Social welfare professionals and other researchers have also urged organizations to proactively engage in key work–family practices to help employees balance their work and family roles [55, 56]. Hence, these social and political forces form various institutional pressures on organizations to adopt family-friendly practices in the workplace. This congruence is likely to be strong for public organizations, which will be more likely to conform to pressure to adopt family-friendly practices.

For example, in the United States, federal and state governments have taken leading roles in promoting childcare and flexible workplace options in the public sector. In 1974, the federal government adopted a flexible scheduling policy, becoming the first major organization to adopt work–family programs [57]. Subsequently, California mandated that all state agencies provide childcare information and referral services to their employees, whereas other states such as Michigan and New York introduced initiatives that fostered public support of childcare for state employees [58]. Similarly, in 2004 approximately 45% of Australian women employed in the public sector had access to paid maternity leave through industrial awards or workplace agreements [59]. Similarly, in Asia, only public employees had access to unpaid parental leave before Taiwan’s government introduced the Gender Equality in Employment Act in 2002 [60]. Using regression analysis on data collected from 993 human resources managers in Spain, Pasamar and Alegre [38] found that normative pressure determined the level of adoption of work–family practices and the use of these benefits by workers. They suggested the need to examine whether an institutional environment conduces work–family balance by adoption of work–family policies and promotes use of these benefits, particularly in the public sector.

H5. Compared to private organizations, the association between public organizations and parents’ WFC will be stronger in countries with more family-friendly leave policy bundles—characterized by (a) higher flexibility of use, (b) longer length of leave, and (c) higher income replacement.
H6. The association between public organizations and parents’ WFC will be stronger in countries with more egalitarian gender roles.

The majority of the literature reviewed here has shown that individuals’ WFC is affected at the macro (social policies) and micro (individuals and families) levels. However, the association between family-friendly policies and WFC among working parents remains unclear. Furthermore, discrepancies in the results of literature examining the effects of gender egalitarianism and gender on WFC could be attributed to differences in methodologies (e.g., only managerial employees, number of participating countries). Some studies found no difference in WFC between men and women [61]. Byron [61] conducted a meta-analysis of 61 published studies that suggested gender has no effects on either WFC or FWC. Due to inconsistencies regarding the moderating effects of gender in the literature, the present study performed statistical analysis separately by gender rather than including it as a control variable to more thoroughly explore similarities and differences between fathers and mothers. Recognizing these gaps in the international work–family literature, the present study examined multilevel effects of family-friendly policies and gender egalitarianism on WFC among working parents by gender, using ISSP data collected in 2005 from national probability samples in 24 countries in Africa, Asia, Europe, and North and South America.

## Method

### Data

This study used the secondary data collected by the International Social Survey Program (ISSP) from random samples of 6,878 employees in 24 countries located in Africa, Asia, Europe, and North and South America to investigate the work–family interface in terms of organizational work–family initiatives, work demands, work–family conflict, and job-related outcomes. The ISSP’s official data archive (https://search.gesis.org/research_data/ZA4350) is the Zentralarchiv at the University of Cologne in Germany. The ISSP is an annual social survey conducted in 40 countries throughout the world. Using a multistage, stratified, probability-proportional-to-size sampling method, the data were collected via a cross-national collaboration in which independent institutions replicated survey questions among random samples in their country.

This study used 2005 ISSP data: Work Orientation modules as publicly available secondary data (de-identified data) that have been stripped of all identifying information and cannot be linked back to the subjects. Data analysis had not involved human subjects; therefore, IRB review was not required for this study. In recent decades, data collected by ISSP have been used widely by other social scholars [11, 13, 27, 45, 62]. The present study used GLOBE’s cultural dimensions of gender egalitarianism as the theoretical perspective [63] that classifies countries into eight clusters: Anglo, Confucian Asia, Eastern Europe, Germanic Europe, Latin America, Latin Europe, Nordic Europe, and Southern Asia. Although the 2005 ISSP data on work orientations were collected from 31 countries, seven countries (i.e., Bulgaria, Cyprus, Czech Republic, Dominican Republic, Flanders, Latvia, and Norway) in the ISSP data were excluded from the analysis because these countries were not included in the GLOBE study.

Based on GLOBE’s theoretical perspective, the 24 countries were ranked based on mean gender egalitarianism practice scores [64]. Hence, ISSP data used in the present study were collected in 24 countries: Australia, Denmark, Finland, France, Germany, Great Britain, Hungary, Ireland, Israel, Japan, Mexico, New Zealand, Norway, the Philippines, Portugal, Russia, Slovenia, South Africa, South Korea, Spain, Sweden, Switzerland, Taiwan, and the United States.

Although ISSP program collected data relevant to work orientation in 1989, 1997, 2005, and 2015, not all 24 countries participated in four-wave data collection. Only 9 countries in western societies participated in the very first wave of data collection in 1989 while South Korea only participated in the 3^rd^ wave of data collection in 2005. In addition, ISSP survey items were somewhat different across 4 waves. Work-family conflict as a variable of interest in this study was not included in the ISSP surveys in 1989 and 1998. It is difficult to pool data from four waves and make cross-national comparisons. Considering that South Korea is a country of research interest for the author, the 2005 ISSP data were used for this study.

Due to hypotheses drawn on theoretical perspectives of this study, the sample was restricted to participants who had children in the household and full-time jobs. Data were collected from one member of each household. A random sample of 6,878 parents (4,015 fathers and 2,863 mothers) was included in the analysis.

### Measurement

#### Dependent variables

*Work–family conflict*. Reflecting a bidirectional conceptualization of WFC, two items were used to measure the degree of stress caused by difficulty balancing work and family domains. Participants were asked whether the demands of their job interfered with their family life (WFC) and whether the demands of their family life interfered with their job (FWC). Responses were scored on a 5-point Likert scale (1 = *always* to 5 = *never*). After reverse coding, higher scores indicated higher levels of WFC or FWC.

#### Independent variables

*Individual-level variables*. ***Organization type***. Organization type was assessed with a single item measuring whether respondents worked for a private or public employer or were self-employed. Responses were coded as 1 = *public sector* and 0 = *private sector or self-employed*.

***Family characteristics***. Marital status (1 = *married*, 0 = *other*) and dual-earner family status (1 = *having a spouse with full-time employment*, 0 = *other*) were dichotomous variables.

***Control variables***. Two covariates, job position and education, were included in the models. Job position (1 = *supervisor*, 0 = *other*) was measured as a dichotomous variable. Education was measured in years.

*Macro-level variables*. ***Family-friendly public policy***. Comprehensive reviews of government-mandated family-friendly policies (i.e., maternity, paternity, parental, and childcare leave) enacted in 2005 in 24 countries were conducted. Because Allen et al. [40] and Moss and O’Brien [9] indicated that national parental leave policies differ in time allotted for leave, flexibility, and percentage of salary paid, three variables were created to measure these three dimensions of family-friendly public policy: flexibility of use, length of leave, and income replacement rate. Qualitative information specified in the parental leave’s statutory statement varies regarding how leave can be used. Thus, based on evaluation criteria suggested by Deven and Moss [65] for reviewing family policies across 19 countries, flexibility of use in this study was measured as 1 = *no flexibility*; 2 = *leave can be taken full-time or part-time*; 3 = *leave can be taken in one block or several blocks of time*; 4 = *leave can be taken for a shorter period with a higher income replacement rate or for a longer period with a lower income replacement rate*; 5 = *leave can be transferred to nonparents*; and 6 = *leave can be taken at any time until a child reaches a certain age* (see Table 1). Higher scores indicated higher levels of freedom with how parents using leave policies and taking time off from work.

**Table 1:** Country-level descriptive statistics (N = 6,878).

Cluster	*n*	WFC		FWC		Flexibility of Use	Length of Leave	IRR (%)	Per Capita GDP
		Fathers	Mothers	Fathers	Mothers				
*Anglo*									
Australia	280	3.15	3.14	2.37	2.42	1	3	0.00	$37,474
Canada	192	2.89	2.93	2.36	2.53	4	3	52.88	$35,093
Great Britain	97	3.26	3.09	2.36	2.16	6	3	20.20	$37,838
Ireland	186	2.69	2.57	2.14	2.01	6	2	35.00	$48,208
New Zealand	243	2.94	2.95	2.13	2.32	3	3	26.92	$27,453
South Africa	396	2.75	2.68	2.34	2.45	1	1	20.00	$5,139
United States	309	2.88	2.54	2.12	2.31	3	1	0.00	$41,553
*Germanic Europe*									
Germany	242	3.09	2.81	2.00	1.97	6	5	39.94	$33,836
Switzerland	164	2.92	2.93	2.65	2.67	1	1	21.54	$50,057
*Latin Europe*									
France	393	2.98	2.88	1.91	2.11	6	5	56.70	$34,152
Israel	228	2.53	2.28	2.00	1.71	1	1	26.92	$20,064
Portugal	420	2.47	2.53	2.03	2.20	6	2	38.81	$18,127
Spain	263	2.43	2.58	1.99	2.15	3	5	31.32	$26,246
*Nordic Europe*									
Denmark	453	2.75	2.70	2.24	2.18	4	3	100.00	$47,567
Finland	238	2.86	2.74	2.25	2.08	3	2	54.04	$37,302
Sweden	229	3.04	3.09	2.46	2.33	6	3	82.19	$40,874
*Eastern Europe*									
Hungary	198	2.68	2.52	1.72	1.67	6	5	103.68	$10,924
Russia	390	2.18	2.41	1.53	1.64	2	5	93.15	$5,340
Slovenia	225	2.87	2.88	1.64	1.70	6	3	105.45	$17,865
*Latin America*									
Mexico	327	2.58	2.64	2.07	2.29	1	1	23.08	$8,033
*Confucian Asia*									
Japan	125	2.38	2.97	2.02	2.43	3	3	66.92	$35,718
South Korea	325	2.24	2.37	1.75	2.09	1	3	50.73	$16,308
Taiwan	625	2.18	2.05	1.89	1.83	3	5	15.38	$15,203
*Southern Asia*									
Philippines	330	2.63	2.81	2.42	2.59	1	1	18.36	$1,156

*Note*. GDP = gross domestic product; IRR = income replacement rate. One-item scale for WFC and FWC ranging from 1 to 5. Flexibility of use was measured as how leave can be used, ranging from 1 = *no flexibility* to 6 = *leave can be taken at any time until a child reaches a certain age*. Length of leave was measured as total statutory postnatal leave, ranging from 1 = *less than 180 days* to 5 = *more than 720 days*. Income replacement rate indicated how well each nation financially supported parents who took leave and was measured as average income replacement provided by each government to each parent who took parental leave during 2005 divided by the per capita gross domestic product of each country.

The unit (e.g., calendar days, weeks, months, years) used for length of leave specified in each country’s written statement regarding statutory parent leave varies greatly. For example, in the United States, the statutory statement sets out 12 weeks for family and medical leave, whereas in South Africa, the statement specifies 4 months for maternity leave. Due to inconsistent units for length of leave ranging from more than 2 months (the Philippines) to 3 years (France) and a lack of the exact numbers of days in most countries’ written statement, a variable was created for total length of leave to enable cross-national comparisons. Length of parental leave (i.e., weeks, months, and years) in certain countries were converted into days. Thus, length of leave was measured as total statutory postnatal leave as 1 = *less than 180 days*; 2 = *between 180 and 360 days*; 3 = *between 361 and 540 days*; 4 = *between 541 and 720 days*; and 5 = *more than 720 days*. Higher scores indicated longer leaves and generosity of the policy.

Moss and O’Brien [9] pointed out that across countries, the format of payments also varies (i.e., flat-rate, mean-tested, paid for only part of the leave period, or a combination of these). A variable for national income replacement rate was created to measure each country’s financial support for parents taking parental leave in 2005. To enable cross-national comparisons, the rate of income replacement indicated how well each nation financially supported parents who took leave, ranging from 15.38% (Taiwan) to 105.45% (Slovenia) rather than the wage replacement (depending on individuals’ income) specified in the statutory statement. Thus, the rate of income replacement was calculated as average income replacement provided by each government to each parent who took parental leave during 2005 divided by the per capita gross domestic product of each country (see Table 1) [66]. Economic development (indicated by per capita gross domestic product) was embedded in the income replacement formula, based on Allen et al.’s [40] meta-analytic study using gross domestic product as an indicator of national economic development, to compare cross-national differences in WFC and FWC across economic contexts.

***Gender egalitarianism***. The present study used GLOBE’s cultural dimensions of gender egalitarianism [63], defined as how societies divided roles between women and men. According to GLOBE’s gender egalitarianism practice score, each country in the present study was classified into one of eight clusters: Anglo, Confucian Asia, Eastern Europe, Germanic Europe, Latin America, Latin Europe, Nordic Europe, and Southern Asia (see Table 1). The Eastern Europe cluster scored highest on gender egalitarianism practice (*M* = 3.84), whereas the Germanic Europe cluster scored lowest (*M* = 3.14). Higher scores indicated more egalitarian gender practices.

### Data analysis

Hierarchical Linear Models (HLM) allowed for multilevel analysis of WFC using individual-level (level-1) and country-level (level-2) variables simultaneously, including tests for cross-level interactions between national family-friendly policies and organization type, marital status, and the effects of dual-earner families on WFC. This allowed for separate error terms and thus, correct estimations of standard errors at both individual and country levels. Hierarchically structured data is nested data where groups of units are clustered together in an organized format. Because the results regarding gender differences in the literature have been inconsistent, data analyses were conducted separately by gender to better understand the influence of gender on the effects of family-friendly policies on WFC (both WFC and FWC). Data analysis strategy follows a certain procedure of HLM to present the results by model. Presenting Model 1 to Model 4 step by step helps the readers understand whether the effects of key individual-level factors differ by country and whether those differences are explained by country-level variables.

The individual-level model was specified as follows:

$$\text{Yij}=\beta 0\text{j}+\beta 1\text{j}*\left(\text{PUBLIC}\right)+\beta 2\text{j}*\left(\text{MARRIED}\right)+\beta 3\text{j}*\left(\text{SPUS}\_\text{FUL}\right)+\Sigma \beta \text{kj}\;\text{Xikj}+\text{rij}$$

in which Yij was the level of WFC or FWC for parent i in country j; β0j was the level-1 intercept; β1j was the effect of public organization; β2j was the effect of marital status (married); β3j was the effect of having a spouse with full-time employment; βkj X were the slopes for k control variables X; and rij was the level-1 error term.

The country-level model was specified as follows:

β0j=G00+G01*(FLEXLEAV)+G02*(LEAVELEN)+G03*(CASHRATI)+G04*(GENEQUAL)+U0j

β1j=G10+G11*(FLEXLEAV)+G12*(LEAVELEN)+G13*(CASHRATI)+G14*(GENEQUAL)+U1j

β2j=G20+G21*(FLEXLEAV)+G22*(LEAVELEN)+G23*(CASHRATI)+G24*(GENEQUAL)+U2j

β3j=G30+G31*(FLEXLEAV)+G32*(LEAVELEN)+G33*(CASHRATI)+G34*(GENEQUAL)+U3j

βkj=Gk

in which G00 was the country-level intercept; G01 was the effect of flexibility of use on the intercept (β0j); G10 was the country-level intercept for the public organization slope; G11–G13 were the cross-level interaction effects of flexibility of use, length of leave, and income replacement rate, respectively, on β1j; G14 was the cross-level interaction effect of gender egalitarianism on β1j; G20 was the country-level intercept for the marital status (married) slope; G21–G23 were the cross-level interaction effects of the three dimensions of policies on β2j; G30 was the country-level intercept for the slope of having a spouse with full-time employment; G3–G33 were the cross-level interaction effects of the three dimensions of policies on β3j; and U0j–U3j were the country-level error terms, assumed to be normally distributed with mean zero and variance σ2. All individual- and country-level variables were centered on their grand means; the intercept indicated average levels of WFC or FWC for men and women in a country with average characteristics.

## Results

### Sample

Table 2 shows individual-level (level-1) descriptive statistics for all 24 countries. Fathers averaged 40.11 years old, compared to 38.38 years old for mothers. Most participants—86% of fathers and 73% of mothers—were married. Thirty-five percent of mothers worked for public organizations compared to 22% of fathers. More fathers were supervisors compared to mothers (42% vs. 29%, respectively). Fathers and mothers received about the same length of education (12.18 years vs. 12.74 years, respectively). Thirty-seven percent of fathers had a spouse with a full-time job, whereas 66% of mothers had a spouse with a full-time job. The average score of WFC was 2.67 for fathers, compared to 2.63 for mothers. The average score of FWC was 2.11 for mothers, slightly higher than FWC scores of fathers (*M* = 2.09).

**Table 2:** Individual descriptive statistics of the sample (N = 6,878).

Variable	Fathers (*n* = 4,015)			Mothers (*n* = 2,863)		
	*M*	*SD*	Range	*M*	*SD*	Range
WFC	2.67	1.08	1–5	2.63	1.08	1–5
FWC	2.09	0.96	1–5	2.11	0.97	1–5
Public organization	0.22	0.41	0–1	0.35	0.48	0–1
Married	0.86	0.35	0–1	0.73	0.44	0–1
Spouse works full-time	0.37	0.48	0–1	0.66	0.47	0–1
Supervisor	0.42	0.49	0–1	0.29	0.45	0–1
Education	12.18	4.33	0–36	12.74	3.74	0–31
Age	40.11	9.27	18–78	38.38	8.97	18–79

Country-level (level-2) descriptive statistics are shown in Table 1. For WFC, British fathers reported highest levels of WFC (*M* = 3.26), whereas Taiwanese fathers had the lowest WFC (*M* = 2.18) among the 24 countries. Australian mothers had highest levels of WFC (*M* = 3.14), whereas Taiwanese women had the lowest WFC (*M* = 2.05). Fathers and mothers in Switzerland experienced the highest levels of FWC (fathers = 2.65, mothers = 2.67), whereas those in Russia had the lowest levels of FWC (fathers = 1.53, mothers = 1.64).

Family-friendly policy bundles—specifically, flexibility of use, length of leave, and income replacement—varied widely across 24 countries. Anglo countries (e.g., Great Britain, Ireland), Latin European countries (e.g., France, Portugal), Eastern European countries (e.g., Hungary, Slovenia), Germanic European countries (e.g., Germany), and Nordic European countries (e.g., Sweden) have implemented highly flexible parental leave policies. Parents in these countries could take leave at any time until a child reached a certain age. In terms of total statutory postnatal leave, France, Germany, Hungary, Russia, Spain, and Taiwan offered more than 2 years. In contrast, Israel, Mexico, the Philippines, South Africa, Switzerland, and the United States had the shortest parental leave, ranging between 67 and 120 days. Except for the United States, the parental leave policy in the latter countries had no flexibility. In terms of income replacement during parental leave, Nordic European countries such as Denmark (100%) and Sweden (82.19%) and Eastern European countries such as Hungary (103.68%), Slovenia (105.45%), and Russia (93.15%) offered high replacement rates, whereas Taiwan (15.38%) and the Philippines (18.36%) had the lowest replacement rates.

### WFC as an outcome variable

Tables 3–5 present the results of four hierarchical linear models of WFC run separately for fathers and mothers. As shown in Table 3, the intraclass correlation (ICC) results indicate that between-country variance constituted 7.2% of the overall variance in WFC among fathers, whereas between-country variance constituted 5.6% of the overall variance in WFC among mothers. The between-country (level-2) variance in the intercept was statistically significant [67] for fathers and mothers in Model 1. Therefore, it was appropriate to examine the multilevel model posited. The intercept showed that fathers had an average WFC score of 2.72, compared to 2.70 for women.

**Table 3:** Hierarchical linear model results for individual- and country-level effects on WFC among fathers and mothers (Model 1).

	Fathers(*n* = 4,015)		Mothers(*n* = 2,863)	
	Coeff.	*SE*	Coeff.	*SE*
Intercept β_0_	2.720*	.061	2.699*	.056
Variance components				
ICC	.072	—	.056	—
Level-2 residual (U_0_)	.085*	.290	.068*	.254
Level-1 residual (*R*)	1.081	1.040	1.098	1.048

*Note*. Level-1 = variables at individual levels such as organization type, marital status, and having a spouse with full-time employment; Level-2 = variables at country levels such as flexibility of use, length of leave, and income replacement.

*p < .05.

**p < .01.

***p < .001.

**Table 4:** Hierarchical linear model results for individual- and country-level effects on WFC among fathers and mothers (Models 2 and 3).

	Model 2				Model 3			
	Fathers		Mothers		Fathers		Mothers	
	Coeff.	*SE*	Coeff.	*SE*	Coeff.	*SE*	Coeff.	*SE*
Intercept β_0_	2.593***	.080	2.630***	.061	2.536***	.080	2.589***	.064
*Individual variables*								
Public organization	-.078*	.037	.085	.051	-.080*	.033	.063	.056
Married	.172**	.049	.026	.069	.138*	.051	.002	.080
Spouse works full-time	-.013	.044	.034	.064	-.018	.044	.029	.066
*Control variables*								
Supervisor	—	—	—	—	.163**	.040	.157**	.044
Education	—	—	—	—	.017**	.005	.022**	.045
Variance components								
Level-2 residual (U_0_)	.119***	.346	.068**	.249	.112***	.335	.067**	.259
Level-1 residual (*R*)	1.073	1.036	1.086	1.042	1.052	1.025	1.063	1.031
*R*^2^ within countries	—	—	—	—	1.96%	—	2.11%	—
*R*^2^ between countries	—	—	—	—	5.88%	—	1.47%	—

*Note*. Coeff. = coefficient. Fathers (n = 4,015); Mothers (n = 2,863)

*p < .05.

**p < .01.

***p < .001.

**Table 5:** Hierarchical linear model results for individual- and country-level effects on WFC among fathers and mothers (Model 4).

	Fathers(*n* = 4,015)		Mothers(*n* = 2,863)	
	Coefficient	*SE*	Coefficient	*SE*
Intercept β_0_	2.543***	.063	2.584***	.051
*Individual variables*				
Public organization	-.089**	.030	.061	.062
Married	.137**	.045	-.003	.081
Spouse works full-time	-.028	.046	.046	.062
*Country variables*				
Flexibility of use	.113**	.039	.069*	.029
Length of leave	-.055	.061	-.069	.050
Income replacement	-1.094**	.363	-.181	.292
Gender egalitarianism	1.050**	.445	.477	.314
*Cross-level interaction*				
Public x flexibility of use	-.052**	.014	.012	.029
Public x length of leave	.079**	.019	-.016	.047
Public x income replacement	.214*	.100	-.005	.327
Public x gender egalitarianism	-.150	.149	-.063	.444
Married x flexibility of use	-.039	.030	-.000	.045
Married x length of leave	-.013	.037	-.018	.056
Married x income replacement	.622	.342	-.083	.495
Married x gender egalitarianism	-.366	.436	.554	.638
Spouse works full-time x flexibility of use	.038	.022	-.0347	.033
Spouse works full-time x length of leave	.080**	.020	.057	.045
Spouse works full-time x income replacement	-.385**	.139	.398	.380
Spouse works full-time x gender egalitarianism	.092	.176	-1.188*	.489
*Control variables*				
Supervisor	.166***	.039	.151**	.045
Education	.017**	.005	.023**	.008
Variance components				
Level-2 residual (U_0_)	.074***	.272	.052*	.227
Level-1 residual (*R*)	1.052	1.026	1.061	1.030
*R*^2^ within countries	.0023%	—	1.88%	—
*R*^2^ between countries	34.16%	—	22.38%	—

*p < .05.

**p < .01.

***p < .001.

Model 2 included the three main individual-level variables—organization type, marital status, and having a spouse with full-time employment. Table 4 shows that working in a public organization had a negative effect on fathers’ WFC (G10 = -.078, *p* = .045). With respect to family characteristics, being married increased levels of WFC among fathers across countries (G20 = .172, *p* = .002), but having a spouse with full-time employment had no effect on WFC among fathers. Conversely, intercept coefficients of the three main family characteristics variables at the individual level had no effect on WFC among mothers.

In Model 3, individual-level control variables were added. For the fathers’ model, coefficients of public organization (G10 = -.080, *p* = .023) and marital status (G20 = .138, *p* = .013) variables were allowed to vary across countries. Job position (G40 = .163, *p* = .001) and education (G50 = .017, *p* = .005) had significant effects on fathers’ WFC; being a supervisor and having higher levels of education increased levels of WFC among fathers. After controlling for job position and education, cross-national differences in the effects of public organization and marital status on WFC among remained significant. All individual-level variables explained 5.9% (.119 versus .112) of the variance in country-level intercepts among fathers. Thus, nearly 6% of the variation in fathers’ WFC across countries was accounted for by cross-national variations in organization type (public) and marital status (being married). However, in the mothers’ model, none of the three main individual-level variables (organization type, marital status, having a spouse with full-time employment) had significant effects on WFC. Instead, job position (G40 = .157, *p* = .002) and education (G50 = .022, *p* = .008) were associated with higher levels of WFC among mothers. All individual-level variables explained 1.5% of the variance in country-level intercepts among mothers.

Model 4 (as shown in Table 5) included the effects of parental leave policies and gender egalitarianism on the intercept and slopes of the three individual-level variables. For fathers, parental leave policies had significant effects on the intercept. Rate of income replacement had a negative effect on the intercept (G03 = -1.094, *p* = .008), whereas flexibility of use had a positive effect on WFC (G01 = .113, *p* = .010). That is, fathers in countries that offered more generous rates of income replacement during parental leave had lower levels of WFC, supporting Hypothesis 3c. In contrast, countries with more flexibility of use regarding parental leave had higher levels of WFC among fathers. Length of leave had no significant effect on WFC among fathers across countries. Therefore, Hypotheses 3a and 3b were not supported. In terms of gender egalitarianism, fathers in countries with more egalitarian gender practices experienced more WFC (G04 = 1.05, *p* = .003). Among mothers, only flexibility of use had a significant effect on WFC (G01 = .069, *p* = .026), suggesting that mothers in countries with more flexible parental leave policies experienced higher levels of WFC.

#### Cross-level interaction

Regarding the moderating effects of parental leave policies on the association between individual-level variables and WFC, flexibility of use (G11 = -.052, *p* = .001), length of leave (G12 = .079, *p* = .001), and rate of income replacement (G13 = .214, *p* = .045) interacted with working for a public organization to predict WFC among fathers. The effects of length of leave and rate of income replacement on working for a public organization were positive, whereas flexibility of use had a negative effect. The results offer support for Hypothesis 5a. For marital status, only rate of income replacement had a moderating effect (G23 = .622, *p* = .085); however, the direction of this effect was in opposition to expectations, which failed to support the hypothesis. In countries offering better rates of income replacement, WFC increased among fathers. With respect to having a spouse with full-time employment, flexibility of use (G31 = .038, *p* = .096) and length of leave (G32 = .080, *p* = .001) had positive moderating effects. That is, in countries with policies that offered more flexible or longer parental leave, fathers with spouses working full-time experienced higher levels of WFC. However, this conflict was reduced by higher rates of income replacement (G33 = -.385, *p* = .013), which supports Hypothesis 5c. Gender egalitarianism had no moderating effect on the individual-level variables among fathers. Adding the three family-friendly policy bundles and gender egalitarianism to Model 4 reduced the between-country variance on the intercept by 34.16% among fathers. In contrast, for mothers, none of parental leave policies had a moderating effect on the association between individual-level variables and WFC. Only gender egalitarianism interacted with having a spouse with full-time employment (G34 = -1.188, *p* = .025). Mothers who had working spouses with full-time employment in countries with more egalitarian gender practices reported lower levels of negative WFC, which supports Hypothesis 2. Adding the three family-friendly policy bundles and gender egalitarianism to the final model reduced the between-country variance on the intercept by 22.4% (from .067 to .052) among mothers.

### FWC as an outcome variable

Tables 6–8 present the results of four hierarchical linear models of FWC run separately by gender. As shown in Table 6, the ICC results indicate that between-country variance constituted 7.6% of the overall variance in fathers’ FWC, whereas between-country variance constituted about 8.1% of the overall variance in mothers’ FWC. The between-country (level-2) variance on the intercept was statistically significant [67] for analysis of variance models for both genders. The intercept showed that the average FWC score among fathers was 2.09, compared to 2.15 among mothers.

**Table 6:** Hierarchical linear model results for individual- and country-level effects on FWC among fathers and mothers (Model 1).

	Fathers(*n* = 4,015)		Mothers(*n* = 2,863)	
	Coefficient	*SE*	Coefficient	*SE*
Intercept β_0_	2.099***	0.055	2.153***	0.058
Variance components				
ICC	0.076	—	0.081	—
Level-2 residual (U_0_)	0.070***	0.290	0.076***	0.276
Level-1 residual (*R*)	0.855	0.924	0.873	0.934

*Note*. Level-1 = variables at individual levels such as organization type, marital status, and having a spouse with full-time employment; Level-2 = variables at country levels such as flexibility of use, length of leave, and income replacement.

*p < .05.

**p < .01.

***p < .001.

**Table 7:** Hierarchical linear model results for individual- and country-level effects on FWC among fathers and mothers (Models 2 and 3).

	Model 2				Model 3			
	Fathers		Mothers		Fathers		Mothers	
	Coeff.	*SE*	Coeff.	*SE*	Coeff.	*SE*	Coeff.	*SE*
Intercept β_0_	1.991***	.065	2.060***	.068	1.973***	.067	2.059***	.069
*Individual variables*								
Public organization	-.017	.032	-.003	.039	-.028	.032	-.025	.043
Married	.128*	.053	.145**	.048	.127*	.054	.166**	.050
Spouse works full-time	.019	.039	-.030	.050	.011	.040	-.046	.052
*Control variables*								
Supervisor	—	—	—	—	.058	.038	-.019	.064
Education	—	—	—	—	.015**	.004	.010*	.005
Variance components								
ICC	.076	—	.081	—	—	—	—	—
Level-2 residual (U_0_)	.075***	.262	.087***	.285	.072***	.269	.084***	.289
Level-1 residual (*R*)	.848	.921	.861	.928	.832	.913	.841	.917
*R*^2^ within countries	—	—	—	—	1.88%	—	2.32%	—
*R*^2^ between countries					4.00%		3.44%	

*Note*. Coeff. = coefficient. Fathers (n = 4,015); Mothers (n = 2,863)

*p < .05.

**p < .01.

***p < .001.

**Table 8:** Hierarchical linear model results for individual- and country-level effects on FWC among fathers and mothers (Model 4).

	Fathers(*n* = 4,015)		Mothers(*n* = 2,863)	
	Coefficient	*SE*	Coefficient	*SE*
Intercept β_0_	1.988***	.059	2.049***	.063
*Individual variables*				
Public organization	-.037	.029	-.022	.036
Married	.097*	.049	.175***	.041
Spouse works full-time	.012	.037	-.036	.047
*Country variables*				
Flexibility of use	.063*	.031	.022	.029
Length of leave	-.066	.041	-.068	.041
Income replacement	-.609*	.269	-.494	.317
Gender egalitarianism	.327	.282	.259	.393
*Cross-level interaction*				
Public x flexibility of use	-.037**	.014	-.054**	.015
Public x length of leave	.071**	.022	-.022	.029
Public x income replacement	.045	.124	.373	.231
Public x gender egalitarianism	.006	.175	-.238	.315
Married x flexibility of use	-.046	.030	.011	.024
Married x length of leave	-.057	.036	.028	.030
Married x income replacement	.574*	.215	-.327	.329
Married x gender egalitarianism	-.478	.243	-.053	.463
Spouse works full-time x flexibility of use	.011	.021	-.066	.027
Spouse works full-time x length of leave	-.002	.025	-.038	.030
Spouse works full-time x income replacement	-.443**	.115	.839**	.251
Spouse works full-time x gender egalitarianism	.310	.166	-.907*	.389
*Control variables*				
Supervisor	.052	.038	-.025	.064
Education	.014*	.005	.011*	.005
Variance components				
Level-2 residual (U_0_)	.066**	.257	.070***	.265
Level-1 residual (*R*)	.832	.912	.840	.916
*R*^2^ within countries	.12%	—	.019%	—
*R*^2^ between countries	8.87%	—	16.19%	—

*p < .05.

**p < .01.

***p < .001

Model 2 included the three individual-level variables: organization type, marital status, and having a spouse with full-time employment. Only marital status had a significant positive effect on FWC among fathers (G20 = .128, *p* = .029) and mothers (G20 = .145, *p* = .007). In other words, married fathers and mothers both experienced higher levels of FWC compared to single parents.

In Model 3, all individual-level control variables were added. The coefficient for marital status remained significant for FWC among fathers (G20 = .127, *p* = .029) and mothers (G20 = .166, *p* = .004). Education was the only control variable with significant effects on levels of FWC for both fathers (G50 = .015, *p* = .006) and mothers (G50 = .010, *p* = .044). Model 4 included effects of parental leave policies and gender egalitarianism on the intercept and slopes of the individual-level variables. The results of Model 4 suggest obvious differences between genders in terms of country-level effects on FWC. For fathers, flexibility of use significantly increased FWC (G01 = .063, *p* = .048), whereas rate of income replacement had a negative effect on the intercept (G03 = -.609, *p* = .036). In countries with more flexible parental leave policies, fathers experienced higher levels of conflict from the family domain to the workplace. In contrast, in countries that offered more generous rates of income replacement, fathers had lower levels of FWC. For mothers, none of the country-level variables had significant effects on FWC.

#### Cross-level interaction

Regarding the moderating effects of parental leave policies on the association between individual-level variables and FWC, flexibility of use (G11 = -.037, *p* = .017) and length of leave (G12 = .071, *p* = .004) interacted with working for a public organization to predict FWC among fathers. The interaction between flexibility of use and public organization was negative, whereas the interaction between length of leave and public organization was positive, rather than negative as expected for Hypothesis 5b. In terms of marital status, rate of income replacement (G23 = .574, *p* = .016) had a moderating effect among fathers. That is, in countries offering better rates of income replacement, married fathers’ levels of FWC conflict increased. Although gender egalitarianism seemed to have a marginal moderating effect (G24 = -.478, p = .064) which assumed that married fathers in countries with more egalitarian gender practices had lower levels of FWC, the result with p value greater than .05 failed to support Hypothesis 2. In terms of having a spouse with full-time employment, rate of income replacement had a significant moderating effect (G33 = -.443, *p* = .001), which supports Hypothesis 4c, whereas gender egalitarianism seemed to have a marginal moderating effect (G43 = .310, *p* = .078) which failed to support the hypothesis. Fathers whose spouse had a full-time were less likely to experience FWC in countries that offered better rates of income replacement which supported Hypothesis 5c. Adding the three family-friendly policy bundles and gender egalitarianism to Model 4 reduced the between-country variance on the intercept by 8.87% among fathers.

For mothers, only flexibility of use interacted with working for a public organization (G11 = -.054, *p* = .003) to predict FWC, as expected for Hypothesis 5a. Women working in public organizations in countries with highly flexible parental leave experienced lower levels of FWC. None of country-level variables interacted with marital status among women. Mothers whose spouse had a full-time job experienced higher levels of FWC (G33 = .839, *p* = .004) in countries that provided more generous rates of income replacement, whereas FWC decreased (G34 = .907, *p* = .031) in countries with more egalitarian gender practices. Adding the three family-friendly policy bundles and gender egalitarianism to the final model reduced the between-country variance on the intercept by 16.19% among mothers.

## Discussion

The present study examined multilevel effects of family-friendly policies and gender egalitarianism on WFC among random samples of working parents across 24 countries. Results show that flexibility of use and rate of income replacement policies during parental leave had significant influences on both WFC and FWC among fathers, whereas only flexibility of use policy had a significant influence on FWC among mothers. The policy with a high rate of income replacement provided by the government while taking parental leave was effective to lessen strain related to negotiating work and family demands among fathers. Surprisingly, parental leave policies with high levels of flexibility of taking leave exacerbated WFC and FWC among fathers. Compared to fathers, the same effect was only found among mothers in terms of WFC.

Brandth and Kvande [68] found that employees in the knowledge professions such as high-tech engineers have a high degree of independence and responsibility in their work, and they often work at home and juggle working hours. Many who are deeply involved in their vocations work beyond normal working hours. Hence, in countries with parental leave policies with greater flexibility of use, the intrusion of work into family life or family life into work may blur the boundaries between the work and family domains and result in higher conflict among both fathers and mothers. This deserves further investigation, especially among professionals in the IT sector. Two studies in the United States and New Zealand illustrated a positive relationship between flexibility of leave use and WFC [69, 70]. These results implied that having greater control over their work schedule strengthened feelings of family life interfering with work among employees, decreasing their ability to fulfill both work and family responsibilities.

Drawn on institutional theory regarding the effect of organization type (i.e., public sector) on WFC, working in public organizations significantly reduced fathers’ WFC but not their FWC. Unlike those in the knowledge professions, which are characterized by flexible work arrangements (e.g., working at home) [68], employees of public organizations with rigid work schedules may be less likely to work at home and have flexible working hours. Therefore, fathers who work for public organizations may be less likely to feel the intrusion of work into family life. Regarding the moderating effects of family-friendly leave policy bundles on the association between organization type and both WFC and FWC, the results show that parental leave policies with high levels of flexibility of use may help fathers working in public organizations decrease their bidirectional WFC. Consistent with institutional theory [40], public organizations are more likely to conform to normative pressure to adopt and support family-friendly practices. Such an institutional environment can help fathers balance their work–family roles. However, for mothers working in the same type of organization, living in countries with more flexible parental leave policies decreased their FWC but not WFC. This gender difference might be due to the traditional role of mothers as major caretakers for the household or child caregivers, as social gender role theoretical perspective posits [33]; therefore, level of flexibility of taking leave allows them to meet family demands in a timely way.

However, findings show that taking longer parental leaves exacerbated conflict between work and family among fathers working in public organizations but not mothers. Fathers may worry about negative consequences on their career prospects, potentially strengthening WFC. Im [71] pointed out that public employees may be required by their employers to assume more work responsibilities and do a better job in less time to maintain efficiency. Absences due to parental leave may cause production loss, costs associated with replacements, or more work for colleagues [72]. Those who take longer parental leave might be stigmatized (e.g., viewed as less productive). However, in this study, the same effect was not found among mothers. Using 2002 ISSP data, Stier et al. [15] found that maternity leave schemes had no effects on working mothers’ inherent conflict between the work of childrearing and paid employment.

Moreover, policies with high rates of income replacement during parental leave period had a greater effect on fathers’ strain in negotiating work and family roles than among mothers. Results showed that policies with higher income replacement rates reduced both forms of WFC among fathers during parental leave. Similarly, a high-income replacement rate policy was found to positively moderate WFC experienced by fathers working in public organizations. Jobs in the public sector tend to have lower pay [73]. As a result, even though public employees receive a high rate of income replacement based on the salary they earn, their lower pay to begin with results in financial setbacks during parental leave periods and has a negative effect on WFC among fathers.

Cross-level interactions between income replacement rate policies and having a working spouse affected fathers and mothers in dual-earner families, albeit in opposite directions. In countries offering policies with high rates of income replacement, fathers in dual-earner families reported less WFC. Having a working spouse who makes financial contributions to the household during the parental leave period may help fathers ease the financial burden of taking leave. In contrast, in countries that offered high rates of income replacement for parental leave, mothers in dual-earner families were more likely to experience FWC. Although women are generally more likely to take parental leave [72], doing so may reduce their opportunities in the labor market. Examining the effects of family policies on income inequality by gender across 20 countries, Mandel and Semyonov [73] found that taking parental leave was costly to women. Taking leave interrupted work continuity and discouraged employers from hiring women for high-status and managerial positions, thereby decreasing their ability to compete successfully with men for the best-paying jobs.

With respect to gender egalitarianism, results of the present study showed that fathers in countries with more egalitarian gender practices experienced greater WFC, contrary to expectations. As gender egalitarianism theory posits [20], men in egalitarian societies are expected to devote more time to family matters and household labor. Therefore, if they spend more time at work, violating societal expectations, men may experience greater WFC. In countries with more egalitarian gender roles, fathers in dual-earner families may be expected to devote more time to family life. However, responding to family needs may interfere with their work.

In contrast, gender egalitarianism negatively moderated the relationship between having spouses with full-time employment and both forms of WFC among mothers. Traditionally, women are more identified with the family domain. They bear a higher burden of household labor and are more likely to be responsible for responding to unique or unplanned family demands, such as leaving work to pick up a sick child from school [74]. Gender egalitarianism theory postulates that when a society is more gender egalitarian, it is less likely to rely on biology to determine gender roles [20]. In such societies, women’s spouse may be expected to assume more family responsibilities despite having full-time employment, easing women’s burden of household labor or family demands. Hence, the present study found that in countries with more egalitarian attitudes regarding gender, mothers who had a spouse who worked full-time were less likely to report WFC or FWC. Campaña et al. [51] suggested that gender equality and work–life balance should be supported through a coherent legislative framework covering maternity leave, paternity leave, parental leave, and caregiver leave, encouraging equal use of leave arrangements by men and women to improve women’s access to and position in the labor market.

Using hierarchical linear models combining information on both individuals and countries, this study showed that parental leave policies had greater influences on WFC among fathers compared to mothers. Individual dimensions of parental leave policies on men’s experience of WFC impinged on workplace characteristics (e.g., organization type) and family characteristics (e.g., having working spouses with full-time employment). Moreover, the data used for the present study have a strong reputation for methodological rigor. This use of cross-national data collected from random samples allows findings to be generalized to populations in countries included in the present study.

Although a solid theoretical framework and cross-national data with probability samples were used, the present study has certain limitations. First, the use of secondary data for analysis has inherent limitations. To better understand the interplay between work and family among dual-earner couples, information on participants’ number of children, occupational prestige, class, workplace size, workplace gender composition, and earlier parental leave-taking should have been collected by the ISSP program. Second, WFC and FWC as dependent variables were each measured by one item in the surveys collected by ISSP program, random measurement error associated with single items may affect accuracy of the results to interpret relationships among variables. Future studies should use validated scales with reliability to measure work-family conflict for analysis. In addition, self-reported survey results might have recollection bias since participants were not identifying a specific situation, precise period, or direct time frame.

Third, variables created for the current study were used to measure three dimensions of family-friendly public policies. Due to inconsistent units for length of leave and various forms of flexibility of use in each country’s statutory statement, these ordinal variables considered as continuous variables (i.e., level of education) were created to make cross-national comparisons. Thus, the results of multilevel analysis should be interpreted with caution. Moreover, certain nuances associated with flexibility of use, length of leave, income replacement rate, and WFC for female employees may have been overlooked.

Fourth, Enders and Tofighi [75] propose that the guideline of centering at the grand mean is appropriate when one is primarily interested in a level-2 predictor and wants to control for level-1 covariates. Thus, this study used centering at the grand mean for all variables. Myers et al. [76] also suggested that the decision regarding centering level-1 predictors in multilevel models should be made based on the research questions, theory, empirical information. Nevertheless, the results of cross-level interactions should be interpreted with caution. Fifth, cultural values of gender egalitarianism used in the present study were based on inferences about national differences in the GLOBE study [64]. It should be noted that individuals in countries vary in values; for example, not all people in an egalitarian society will have egalitarian attitudes toward gender. Sixth, the data come from a cross-sectional wave of the ISSP project; therefore, causal relationships between independent and dependent variables relevant to the results of this study cannot be inferred. Although ISSP program collected data with the same theme topic (work orientation) at four time points (1989, 1997, 2005, 2015), survey items were somewhat different across 4 waves. For example, work-family conflict as a variable of interest in this study was not included in the ISSP surveys in 1989 and 1998. In addition, participated subjects were not the same ones across 4 waves and many countries hadn’t participated in four-wave data collection. Thus, given ISSP research design with certain limitations it is challenging using the data for a longitudinal study focusing on work-family conflict with cross-national comparison.

Finally, in their meta-analytic findings derived from reviewing 332 studies published between 1992 and 2018, Allen et al. [4] underscored a need for future studies to examine institutional policies as a factor that could explain between-country differences in work–family experiences in the context of culture and economy. Using the same ISSP data from 2005, Ruppanner and Huffman [7] suggested more empirical studies should use multilevel investigations in a cross-national context. Although the ISSP data used in this study were more than a decade old, results derived from the research model using random probability samples of working parents across 24 countries may provide implications for policy makers and employers in public and private sectors in developing countries featuring an increasing rate of labor force participation among women (e.g., India, Vietnam), traditional gender attitudes, certain workplace characteristics (e.g., electronics and garment factories), and family structures similar to those of the past decade in developed countries. The findings of this study may further the improvement of family-friendly policies, development of work–family theoretical models, and meta-analytic research in cross-cultural contexts with greater explanatory power.

### Implications for research and practice

The findings of the present study suggest the importance of considering workplace context and gender roles when implementing family-friendly policies. The results indicate that both fathers and mothers were likely to face costs associated with taking leave from work, which deserves attention in future research. Workplace characteristics influenced fathers more than mothers in terms of the effects of parental leave flexibility of use, length of leave, and income replacement rates on WFC. Negative consequences may be associated with absence from the workplace, especially among women. More research is needed to investigate relationships among workplace gender composition and size to reveal the mechanisms that link policy, work characteristics, and WFC. Furthermore, WFC among men and women may be significantly influenced by earlier parental leave-taking by colleagues, workplace-specific social networks, and hierarchies in the workplace. Family policies such as maternity and paternity leave might be different for salaried and self-employed workers. For example, do self-employed mothers use their time differently than their salaried counterparts? Researchers could explore whether there are some specific characteristics of leave policies (e.g., leave being full time or part time) that might reduce work-family conflict and the mechanisms that link parental leave policies, WFC, and consequences of taking leave to provide more nuanced information about how women may be negatively affected by taking a leave of absence.

The main objectives of family-friendly policies are to reduce the conflict between family roles and commitment to work, allow more women to join the labor force, and improve the fertility rate. The findings of present study suggest that implementing parental leave policies with high flexibility and higher rates of income replacement may help fathers with a working spouse or who are employed in the public sector to better balance their work and family demands. In this study, mothers generally were not protected by individual dimensions of parental leave policies across countries. Instead, societal attitudes toward gender played a key role in helping mothers reduce bidirectional conflicts between work and family roles.

Findings of this study provide implications for policy makers and employers, especially those in developing countries (e.g., Vietnam, India) characterized by increased involvement of women in the job market with traditional workplace characteristics, related to guiding their family-friendly leave policies to cater to the specific needs of women. Gender egalitarianism might be best achieved if both parents have access to workplace supports such as childcare that allow them both to seamlessly work long hours. To promote work–life balance among working parents, policy makers and regulators need to monitor and examine diffusion and patterns of parental leave use in the workplace by gender and evaluate the effectiveness of parental leave policies for policy implementation and improvement.
